# Gaussian diffusion sinogram inpainting for X-ray CT metal artifact reduction

**DOI:** 10.1186/s12938-016-0292-9

**Published:** 2017-01-05

**Authors:** Chengtao Peng, Bensheng Qiu, Ming Li, Yihui Guan, Cheng Zhang, Zhongyi Wu, Jian Zheng

**Affiliations:** 1 Center for Biomedical Engineering, Department of Electronic Science and Technology, University of Science and Technology of China, Hefei, China; 2 Medical Imaging Department, Suzhou Institute of Biomedical Engineering and Technology, Chinese Academy of Science, Suzhou, China; 3 PET Center, Huashan Hospital, Fudan University, Shanghai, China

**Keywords:** Metal artifact reduction, Sinogram inpainting, Gaussian diffusion, Prior image, X-ray CT

## Abstract

**Background:**

Metal objects implanted in the bodies of patients usually generate severe streaking artifacts in reconstructed images of X-ray computed tomography, which degrade the image quality and affect the diagnosis of disease. Therefore, it is essential to reduce these artifacts to meet the clinical demands.

**Methods:**

In this work, we propose a Gaussian diffusion sinogram inpainting metal artifact reduction algorithm based on prior images to reduce these artifacts for fan-beam computed tomography reconstruction. In this algorithm, prior information that originated from a tissue-classified prior image is used for the inpainting of metal-corrupted projections, and it is incorporated into a Gaussian diffusion function. The prior knowledge is particularly designed to locate the diffusion position and improve the sparsity of the subtraction sinogram, which is obtained by subtracting the prior sinogram of the metal regions from the original sinogram. The sinogram inpainting algorithm is implemented through an approach of diffusing prior energy and is then solved by gradient descent. The performance of the proposed metal artifact reduction algorithm is compared with two conventional metal artifact reduction algorithms, namely the interpolation metal artifact reduction algorithm and normalized metal artifact reduction algorithm. The experimental datasets used included both simulated and clinical datasets.

**Results:**

By evaluating the results subjectively, the proposed metal artifact reduction algorithm causes fewer secondary artifacts than the two conventional metal artifact reduction algorithms, which lead to severe secondary artifacts resulting from impertinent interpolation and normalization. Additionally, the objective evaluation shows the proposed approach has the smallest normalized mean absolute deviation and the highest signal-to-noise ratio, indicating that the proposed method has produced the image with the best quality.

**Conclusions:**

No matter for the simulated datasets or the clinical datasets, the proposed algorithm has reduced the metal artifacts apparently.

## Background

The use of X-ray computed tomography (CT) in the clinical setting has lasted for several decades and has considerably helped disease diagnosis and therapy. However, metallic implants in the bodies of patients usually produce severe streaking artifacts, which will obscure crucial information and reduce image quality. The academic community believes the explanation of this phenomenon is beam hardening [1, 2]. Due to the pleochroism of the X-ray energy spectrum, the attenuation coefficient of the high energy X-ray is smaller than the low energy X-ray. That is to say, when an X-ray passes through body of a patient containing metal, the low energy photons are absorbed and output mostly contains high-energy photons, thus the high-energy photons detected by detector become richer. Obviously, the conventional algorithm that is widely used in CT image reconstruction, namely the filtered back-projection (FBP) reconstruction algorithm [3], fails to reconstruct the image clearly in this situation due to inaccurate projection data. To solve the problem of severe streaking artifacts, many metal artifact reduction (MAR) algorithms have been proposed.

Presently, the most commonly used MAR algorithm is the interpolated sinogram inpainting MAR algorithm, aimed to interpolate missing projection data using neighboring data by replacing the corrupted data from the information of nearby slices and then using FBP to reconstruct the image. This type of algorithm has an appealing merit in its computation speed. Kalender et al. [4] have proposed to interpolate corrupted projection data via linear interpolation (LI) from their neighbors. The problem is that the transition between the original and interpolated projections is not sufficiently smooth, causing severe secondary artifacts. In addition to LI, cubic spline [5, 6], wavelet interpolation [7], and total variation [8–10] sinogram inpainting techniques are also used in MAR. Meanwhile, other methods try to replace the corrupted projections by relying on nearby regions or corresponding prior projections. For example, Chen et al. [11] proposed to restore corrupted projections using nearby regions via the similarity criterion. First, they segmented implanted metals and metal artifacts from the original image. Second, they restored the corrupted projections relying on nearby projections via the similarity criterion. The results of this method are largely dependent on the quality of the segmentation. However, when the metal artifacts contain complicated bone structures, this approach is likely to perceive the bone as bright artifacts, therefore causing a blurring of the bone structures in the corrected image. Tang et al. [12] restored the corrupted projections by replacing the metal slices with corresponding prior projections. In this method, the prior image is reconstructed with a down-sampled sinogram. This method sacrifices high-frequency details so that the final corrected image is relatively vague, and the details of the tissue cannot be assessed. Like Tang et al., Bal and Spies [13] have proposed to replace the metal slices in the projection domain by prior images in the same region. The problem with this method is that the prior projections over the metal trace are not well-fitted with their neighbors in the originals, thus producing new artifacts. To solve the problem of fitness in the interpolated sinogram inpainting technique, Meyer et al. [14] have proposed the normalized metal artifact reduction (NMAR) algorithm. They first normalized the original sinogram using a prior sinogram, the corrupted data were linearly interpolated and the final completed sinogram was obtained by de-normalizing the interpolated sinogram. Because the NMAR algorithm still relies on LI, when used in an image with complicated structures, such as a tooth image, secondary artifacts would increase. Additionally, in the NMAR algorithm it is hard to avoid severe streaking secondary artifacts when the target contains more than one metal because it cannot interpolate the metal trace accurately.

Based on the forward sinogram inpainting MAR framework, in this paper we propose a Gaussian diffusion sinogram inpainting technique based on a prior image to reduce metal artifacts in a Bayesian framework. In this work, the optimization target is to minimize prior energy. To recover an uncorrupted image from an incomplete dataset, the sparsity of the target image is improved by subtracting the prior image. Additionally, the Gaussian diffusion function is applied to diffuse energy to recover missing projections. The Gaussian diffusion process is realized via a gradient descent algorithm. The final target image is reconstructed using the FBP algorithm. Compared with the algorithms listed in the last paragraph, this proposed method does not show the smoothness problem and can be effective for CT images that contain several metal objects. In this work, the performance of the proposed algorithm is compared with the LI and NMAR algorithms using both simulation and clinical studies. The evaluation criteria include image quality and quantitative analysis within the region of interest, all of which demonstrate that our method performs better than the other two MAR algorithms.

## Methods

### Problem formulation and optimization approach

Let *x* denote the uncorrupted projection dataset, let *y* represent the dataset with the metal projection data removed, let Ω represent the metal trace set (metal-corrupted projection region), and let *n* represent an unknown noise. We can then formulate the following model:

1 $$y = Hx + n$$

where *H* is an operator that removes the metal-corrupted projections from *x*.

In the classical least squares (LS) approach [15, 16], the estimator is chosen to minimize noise:

2 $$\left( {\text{LS}}\right):\;\hat{x}_{LS} = \mathop {\arg \hbox{min} }\limits_{x} \left\| {y - Hx} \right\|^{2}$$

However, in this work the matrix *H* is ill-conditioned, thus the LS solution usually has a huge norm and is meaningless. We then follow the Bayesian approach [17] whose optimization target in this work is to maximize the posterior probability of *x* given *y*, which is defined as:

3 $$P\left( {x/y} \right) \propto P\left( {y/x} \right)P\left( x \right)$$

*P*(*y*/*x*) is defined as:

4 $$P\left( {y/x} \right)\sim\,e^{{ - \left\| {y - Hx} \right\|^{2} }}$$

In this framework, the target *x* is regarded as a stochastic quantity with a prior density, and:

5 $$P\left( x \right)\sim\,e^{ - U\left( x \right)}$$

where *U*(*x*) is the prior energy. Actually, this imposes the prior information onto the estimation. Now, Eq. (3) can be reformulated as follows:

6 $$P\left( {x/y} \right)\sim\,e^{{ - \left( {\left\| {y - Hx} \right\|^{2} + U\left( x \right)} \right)}}$$

Next, the optimization target is to minimize the posterior energy, which is formulated as:

7 $$\hat{x} = \mathop {\arg \hbox{min} }\limits_{x} \left( {\left\| {y - Hx} \right\|^{2} + U\left( x \right)} \right)$$

In this formulation (7), the first part measures the difference between *x* and *y*, and the second part restricts *x* to the prior information. In this work, with the decrease of noise, *y* is approximately equal to *Hx*, so the first term in (7) is close to zero. Next, the problem defined in (7) is redefined as a constrained optimization problem:

8 $$\hat{x} = \mathop {\arg \hbox{min} }\limits_{x} U\left( x \right),\quad subject\;to\;y = Hx$$

To solve the problem of (8), we bring in the diffusion equation: [16]

9 $$I_{s} = f\left( s \right) \cdot {\nabla }I$$

where the symbol ∇ is a derivative matrix, formed by the first-order forward difference in this work. *I* represents the diffusion target, and *I*_*s*_ is the result after diffusion. *f*(*s*) is a Gaussian diffusion function: [19, 20]

10 $$f\left( s \right) = e^{{ - \frac{{s^{2} }}{{2\delta^{2} }}}}$$

where *s* = ||∇*x*_*p*_||, *x*_*p*_ is the sinogram of the prior image, called a prior sinogram. The parameter *δ* determines the level of diffusion.

According to [18], the diffusion process can be observed as a gradient descent of the prior energy, and the gradient descent algorithm is:

11 $$- \lambda U^{{\prime }} \left( x \right) = - \lambda {\nabla }^{T} \left( {f\left( {\left\| {{\nabla }x_{p} } \right\|} \right)} \right){\nabla }\left( {x - \mu x_{p} } \right)$$

where the parameter *μ* weighs the effect of the prior sinogram, and *λ* is the descent speed factor.

In Eq. (11), we introduce *x*_*p*_ for two reasons: first, to improve the sparsity of the target sinogram; second, to obtain the diffusion objective.

After the step of gradient descent, the algebraic reconstruction technique (ART) is applied to reduce the projection onto the convex set (POCS): [21]

12 $$proj\left( {\tilde{x}} \right) = \tilde{x} + H\left( {x_{ori} - \bar{x}} \right)$$

where *x*_*ori*_ is the original sinogram, and $\bar{x}$ is the sinogram after the step of gradient descent.

In Table 1, *x* ^−1^ and *x* ^0^ are set equal to *x*_*ori*_, the step size *λ* is set to 0.03 and *δ* = 4, according to our experience and numerous experiments. The matrix *H* is exploited to remove the metal trace, and it is constructed according to the position of the missing projections: the value in the position of the metal in the projection domain is set to zero, and the value in other areas is set to one. What calls for special attention is that the operator *H* multiplies others is multiplying the corresponding elements between two matrices. Step 1 and step 2 are quoted from the literature [16]. The stopping criterion of the iteration is defined when the relative difference between *x* ^*k*+1^ and *x* ^*k*^ blows the tolerance (||*x* ^*k*+1^ − *x* ^*k*^||/||*x* ^*k*^|| < *η*, *η* = 1 × 10^−4^) [16, 17].

**Table 1:** The Gaussian diffusion sinogram inpainting MAR algorithm

Initialize *k* = 0, *η* = 1×10^−4^, *μ* = 1, *δ* = 4, *t* ^0^ = 1.
Do {
Step 1. $$t^{k + 1} = \left( {1/2} \right)\left( {1 + \sqrt {1 + 4\left( {t^{k} } \right)^{2} } } \right).$$
Step 2. $$\bar{x} = x^{k} + \left( {t^{k} - 1} \right)/t^{k + 1} \left( {x^{k} - x^{k - 1} } \right).$$
Step 3. $$\tilde{x} = \bar{x} - \lambda {\nabla }^{T} f\left( {\left\| {{\nabla }x_{p} } \right\|} \right){\nabla }\left( {x^{k} - x_{p} } \right).$$
Step 4. $$x^{k + 1} = \tilde{x} + H\left( {x_{ori} - \tilde{x}} \right).$$
Step 5. *k* = *k*+1.}
While $$\left( {\left\| {x^{k + 1} - x^{k} } \right\|/\left\| {x^{k} } \right\| > \eta } \right){.}$$

**Fig. 1:**
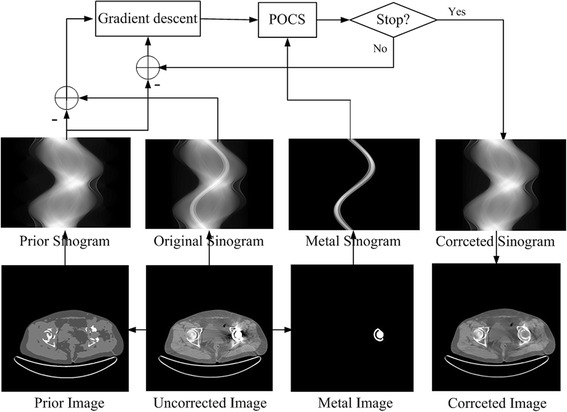
Diagram of the proposed Gaussian diffusion sinogram inpainting algorithm. From the uncorrected image, the prior image and metal-only image are obtained. The corresponding sinograms are yielded via forward projection. The gradient descent algorithm and POCS process are applied to restore the sinogram. The final corrected image is reconstructed via the FBP method from the corrected sinogram

Figure 1 provides the diagram of the proposed algorithm. In this diagram, the corresponding sinograms are produced by forward projection. Using a simple thresholding technique, the metal-only image is obtained, and the CT value for the metal is set to 3000 HU.

Concerning the prior image, Li et al. proposed an image post-processing strategy to create the prior image. First, they segmented the metal, and then a prior image was produced via an edge-preserving filter and a recovery procedure of the adjacent anatomical structures [22]. Meyer et al. [14] applied a simple thresholding method to segment air, soft tissue, and bone after the image was filtered by Gaussian. Next, the CT values for a portion of air and soft tissue were set to −1000 and 0 HU, respectively. Bone pixels kept their values because of the inherent variability of the bone densities. Different from Bal and Spies [13] who set the values of air and soft tissue with their own average CT numbers, in this work we obtained the prior image by the method of [14].

### Experiment materials

The performance of the proposed algorithm is tested on both the simulated and clinical datasets, and the results are compared with two conventional algorithms (LI and NMAR). In the simulated studies, the jaw and hip phantom are applied, and the phantom size is 200 mm × 200 mm. In the simulation, we suppose that the process of the detector receiving photons can be modeled by the Poisson distribution [23]:

13 $$I_{i} = Poisson\left\{ {I_{0} \cdot \exp \left( { - \int_{{L_{i} }} {u\left( {l,E_{k} } \right)dl} } \right) + S_{k}^{i} } \right\} + Normal\left( {0,\delta_{e}^{2} } \right)$$

where *I*_*i*_ is the number of photons received by the detector, *I*_0_ is the number of photons emitted, *L*_*i*_ is the X-ray path, *u* is the attenuation coefficient of tissue, $S_{k}^{i}$ is the statistical scattering photon number, and $\delta_{e}^{2}$ is the noise variance. After logarithmic processing for the result of (13), the projection that contains noise and beam hardening is obtained. In this work, we set *E*_*k*_ = 60 keV, *I*_0_ = 5.0 × 10^5^ for the hip phantom and *I*_0_ = 5.0 × 10^6^ for jaw phantom, $S_{k}^{i} = 150$, $\delta_{e}^{2} = 10$.

The clinical dataset includes four patients: a patient with a single metal tooth, a patient implanted with two metal teeth, a patient with a single hip prosthesis and a patient with multiple metals implanted in the vertebrae.

In this work, to acquire artificial projection data under conditions close to the actual acquisition, we consider the fan-beam geometry of the simulated single-slice CT scanner with 1024 detector channels, 720 angular samples over an 360° orbit, and the CT image resolution is 512 × 512 pixels. All of the CT images are processed on a PC workstation (Intel Core 3 CPU 3.60 GHz processor and 4096 Mb RAM) by MATLAB 2010.

### Evaluation criteria

The performance of the Gaussian diffusion sinogram inpainting algorithm was compared with the LI and NMAR algorithms subjectively and objectively. We subjectively evaluated the image quality for the proposed algorithm and the two other compared algorithms. We objectively evaluated the performance using the signal-to-noise ratio (SNR) and the normalized mean absolute deviation (NMAD) for the corrected images:

14 $$SNR = 10\lg \left( {\frac{{\sum\nolimits_{i,j} {\left( {u_{i,j}^{truth} } \right)^{2} } }}{{\sum\nolimits_{i,j} {\left( {u_{i,j} - u_{i,j}^{truth} } \right)^{2} } }}} \right)$$

15 $$NMAD\left( \% \right) = 100 \times \frac{{\sum\nolimits_{i,j} {\left| {u_{i,j} - u_{i,j}^{truth} } \right|} }}{{\sum\nolimits_{i,j} {\left| {u_{i,j}^{truth} } \right|} }}$$

where *u*_*i*,*j*_ represents the corrected image, and $u_{i,j}^{truth}$ is the ideal image. Generally, SNR measures the anti-noise performance of the algorithm, and its value is greater, the algorithm produces a better quality image; NMAD measures the difference between result image and ideal image, and its value is smaller, the algorithm produces a result that is much closer to the ideal image. Besides, the mean pixel value and the standard deviation of different region of interests (ROIs) in each resulted image are analyzed.

For the clinic datasets, the ROI is defined on uncorrected images containing the metallic implants, and it is magnified for observing clearly. Besides, the reference images of the clinic datasets is hard to define, thus, we evaluated the performance only by the image quality.

## Results

In this section, the results corrected by the LI, NMAR and proposed algorithms for the simulation and clinical datasets are presented. We first analyzed the simulated results, and the clinical part was analyzed subsequently.

**Fig. 2:**
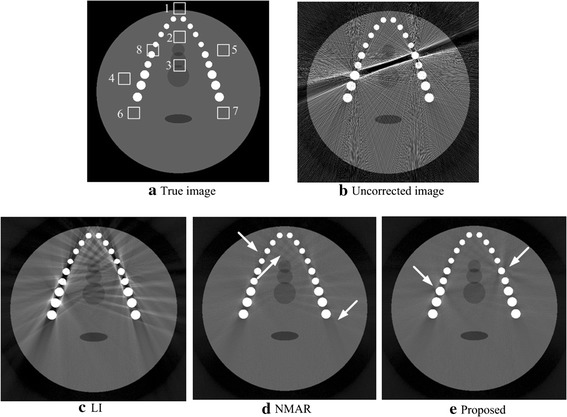
Images of the jaw phantom. **a** Jaw phantom without any artifacts. **b** Uncorrected images with severe streaking artifacts. **c**–**e** The results corrected via LI, NMAR and the proposed algorithms. The display window is [0 0.53]

Figure 2 shows the results for the jaw phantom with two metal dental fillings. Figure 2a is the true image, which contains no artifacts. Figure 2b is the uncorrected image with severe streaking metal artifacts. However, Fig. 2c–e are the corrected images produced by LI, NMAR and the proposed algorithm. Clearly, the original metal artifacts are successfully reduced by each method. Unfortunately, the LI MAR algorithm causes severe secondary artifacts, which blur the tissue around the metal. NMAR and the proposed algorithms reduce the artifacts with fewer secondary artifacts than LI, and with regard to the entire picture, the proposed algorithm has a better result than NMAR, especially in the area between the teeth (as indicated by the arrows in Fig. 2d). However, in the proposed algorithm result, some slight artifacts are introduced around the metals, indicated as arrows in Fig. 2e primarily because the prior image was not adequate.

Table 2 shows the quantitative evaluation of experimental results. From the table, the NMAD of the proposed algorithm is smaller than those of the LI and NMAR algorithms, indicating that the image corrected by the proposed algorithm is closer to that of the true image. While the largest SNR indicates that the proposed algorithm has a better effect on suppressing the metal artifact and noise, thus producing a higher quality image.

**Table 2:** Quantitative evaluation of the experimental results for the jaw phantom

Uncorrected		LI		NMAR		Proposed	
SNR (dB)	NMAD (%)	SNR (dB)	NMAD (%)	SNR (dB)	NMAD (%)	SNR (dB)	NMAD (%)
12.14	57.27	22.97	14.37	27.23	9.49	27.54	9.34

**Fig. 3:**
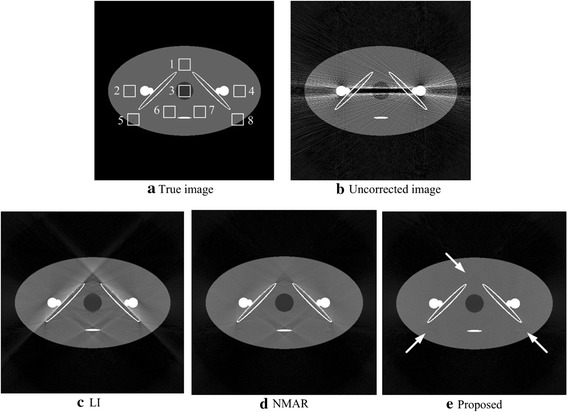
Images of the hip phantom. **a** The hip phantom without any artifacts or noise. **b** The uncorrected image with severe streaking artifacts. **c**–**e** The results corrected via the LI, NMAR and proposed algorithms. The display window is [0 0.52]

Figure 3 compares the results for the hip phantom with a double-metal prosthesis. Figure 3a is the reference image with no artifacts and noise. Figure 3b is the uncorrected image, as observed the severe beam harden artifacts between the metals. Figure 3c–e are the images corrected by the LI, NMAR and proposed algorithms. All of the MAR algorithms have a good effect on reducing the metal artifacts, while the LI method introduces many residual artifacts and blurs the bone structures. Compared with LI, NMAR has a better quality image but some residual artifacts persist. Regarding the proposed algorithm, the image only introduces slight secondary artifacts, which are indicated by the arrows in Fig. 3e. Thus, from subjective judgment, the proposed algorithm performs the best.

Table 3 gives the quantitative evaluation of the hip phantom results. In this table, the proposed algorithm NMAD is smaller than LI and NMAR, decreasing by 2.93 and 0.51%, respectively, revealing that the image corrected via the proposed algorithm has a better representation for the true image compared with the other two algorithm results. The SNR of the proposed algorithm is larger than LI and NMAR,increasing by 9.35 and 1.04 dB, respectively, implying the proposed algorithm leads to the best corrected image among the three MAR algorithms.

**Table 3:** Quantitative evaluation of the experimental results for the hip phantom

Uncorrected		LI		NMAR		Proposed	
SNR (dB)	NMAD (%)	SNR (dB)	NMAD (%)	SNR (dB)	NMAD (%)	SNR (dB)	NMAD (%)
12.91	56.05	13.34	18.04	21.65	15.62	22.69	15.11

**Fig. 4:**
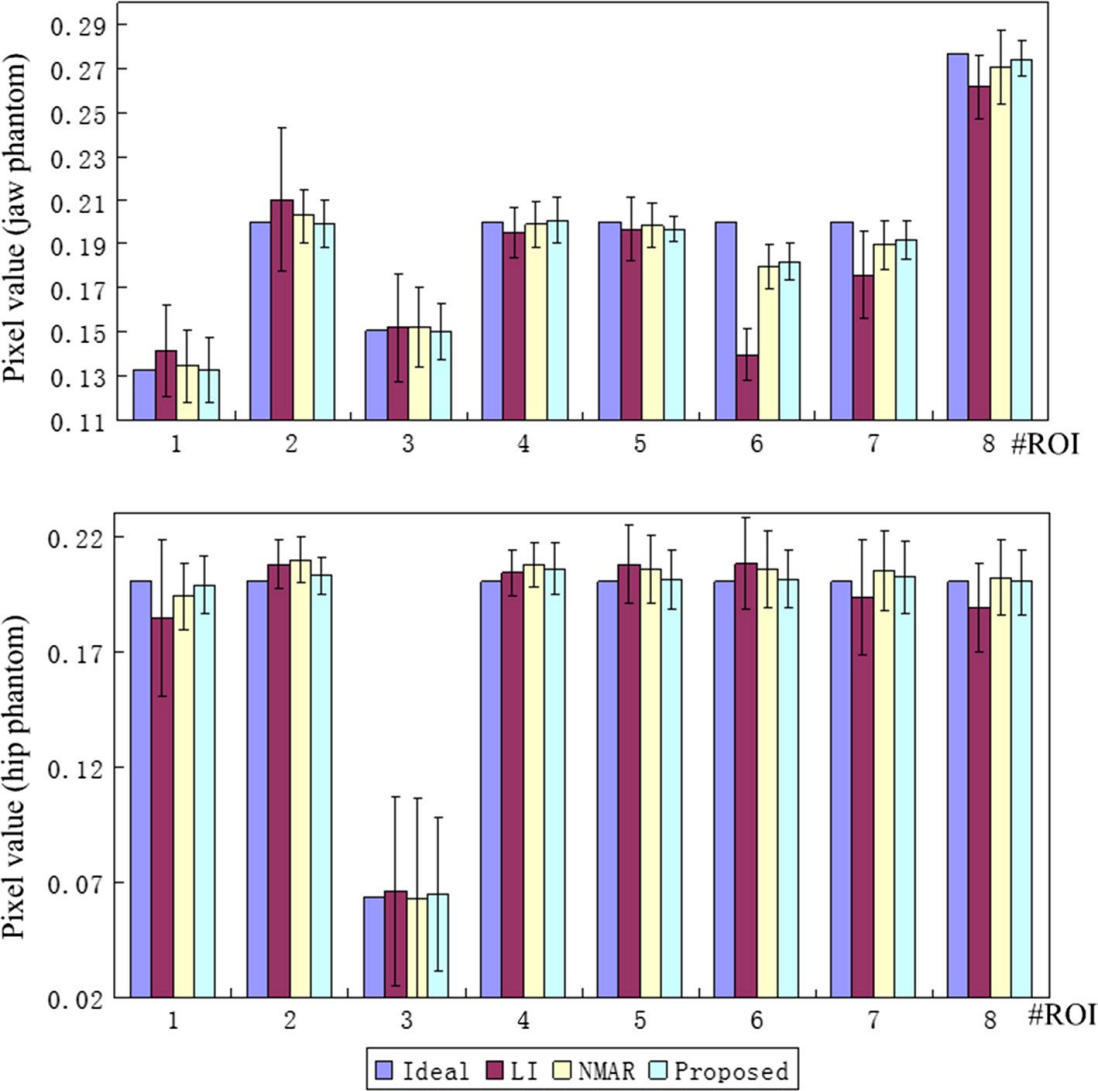
The mean pixel value and the standard deviation in different ROIs for the two phantoms. The *top* one are the results of the jaw phantom and the *bottom* one belong to the hip phantom. *Eight* RIOs are defined in Figs. 2a and 3a for two phantoms, and each ROI contains 20 × 20 pixels

Figure 4 shows the mean pixel value and the standard deviation of eight ROIs in resulted images for the two phantoms. The ROIs of different phantoms are defined in Figs. 2a and 3a. Generally, the proposed method has the mean pixel that is closest to the ideal, and it also has the smallest standard deviation in most ROIs. The results imply that the proposed algorithm has the best performance in suppressing the artifacts.

These two groups of phantom experiments show that whether the bone structure in the image is simple or complex, the proposed algorithm has a good effect on suppressing metal artifacts and reducing the noise level.

**Fig. 5:**
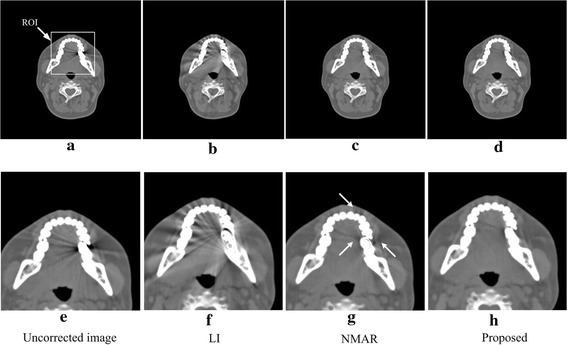
Uncorrected image and results corrected via the different algorithms for the patient with a single metal dental filling. **e**–**f** The local amplifications of the corresponding images (**a**)–(**d**). The display window width and window center are 1200 and 100 HU, respectively

Figure 5 illustrates the correction results for the patient with a single metallic dental filling. Figure 5a is the uncorrected image. Figure 5b–e are the corrected images produced by the LI, NMAR and proposed algorithms. Figure 5e–h are the images after local amplification of the corresponding Fig. 5a–d in the ROI; this operator is beneficial for observing the image quality in ROI clearly, and the ROI is defined in Fig. 5a with a white rectangle. In the LI MAR result, the image shows severe secondary artifacts, and some structures are even more blurred. Both NMAR and the proposed algorithm successfully reduce the artifacts and introduce hardly any secondary artifacts, but the proposed algorithm performs better. As indicated by the arrows in Fig. 5g, the residual dark artifacts of the NMAR result are more than those of the proposed one. Thus, in this dataset, the proposed method performs the best, and the performance has proven that the algorithm can be used in complicated bone structure images containing a single implanted metal.

**Fig. 6:**
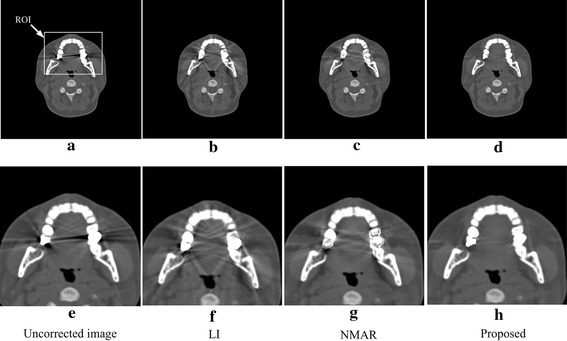
Uncorrected image and results corrected via the different algorithms for the patient with double-metal dental fillings. **e**–**f** The local amplifications of the corresponding images (**a**)–(**d**). The display window width and window center are 1600 and 300 HU, respectively

Figure 6 shows the corrections for the patient with two metallic dental fillings. Figure 6a–d are the uncorrected images and results corrected via the LI, NMAR and proposed method. Figure 6e–h are the local amplifications of the corresponding Fig. 6a–d in the ROI, and the ROI is defined in Fig. 6a by the white rectangle. In this dataset, the LI reduces the original metal artifacts existing in the uncorrected image but introduces severe secondary artifacts, making the image quality even lower than that of the uncorrected image. Compared with LI, NMAR produces a better result and does not cause artifacts between teeth; however, secondary artifacts still blur the structures between the two metals. While the result produced via the proposed algorithm reduces the metal artifacts without causing any obvious secondary artifacts, the tissue structures between the two metals and around the teeth are very clear. Thus, in this dataset, the proposed method has the best performance, demonstrating that the proposed algorithm has a good effect on reducing the artifacts under the condition of two metal fillings in a complicated bone structure image.

**Fig. 7:**
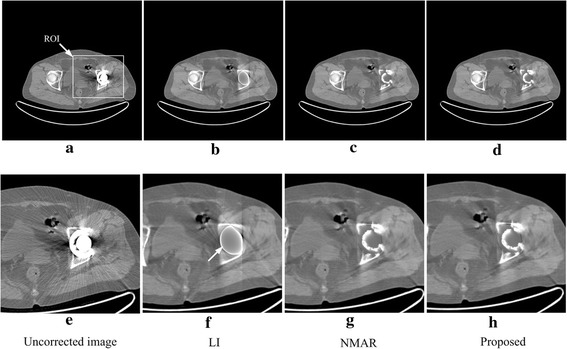
Uncorrected image and results corrected via the different algorithms for the patient with a single metallic hip prosthesis. **e**–**f** The local amplifications of their corresponding images (**a**)–(**d**). The display window width and window center are 800 and 0 HU, respectively

Figure 7 shows the correction results for the patient with a single metallic hip prosthesis. Figure 7a is the uncorrected image, and Fig. 7b–d are the results corrected via LI, NMAR and the proposed methods. Figure 7e–h are local amplifications of the corresponding Fig. 7a–d in the ROI; the ROI is defined in Fig. 7a by the white rectangle. In this dataset, to analyze the quality of the images produced by these MAR methods, we have not implanted metal into the results. For the LI result, as indicated by the arrow in Fig. 7f, the region around the metal is totally blurred; in addition, some secondary artifacts are also introduced. Altogether, for the results from the NMAR and proposed methods, they both reduce metal artifacts thoroughly and cause no new artifacts. There are two main reasons for this. First, both the NMAR and proposed methods use the same prior image, implying the proposed method relies on the prior image to some extent. Second, the uncorrected image has a simple structure, which determines that the metal artifacts is easy to be corrected. On the other hand, due to the uncertainty of the prior image with the method of [14], the prior image of this experiment just makes the NMAR have the same good result as the proposed algorithm. The combination with the previous three groups of experimental results of the NMAR shows that the proposed method is more stable and lower requirement for prior image quality compared to the NMAR. In the next dataset, there are complex bones and soft tissues in the uncorrected image, indicating that the correction of this dataset is the most challenging.

**Fig. 8:**
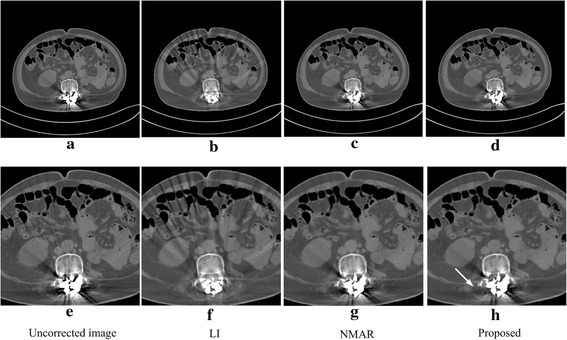
The uncorrected image and the corrected results via the different algorithms for a patient with multiple metallic implants in the vertebrae. **e**–**f** The corresponding images after amplifying (**a**)–(**d**) 1.6 times. The display window width and window center are 800 and 100 HU, respectively

The correction results from the most challenging dataset are shown in Fig. 8. This dataset is from a patient whose vertebrae are implanted with several metals. Figure 8a is the uncorrected image, which shows severe streaking dark artifacts around the metals and tissue structures that are very complicated, indicating that the process of MAR is not easy. Figure 8b–d are the results corrected via the LI, NMAR and proposed methods. After magnifying Fig. 8a–d 1.6 times, the corresponding images are obtained, Fig. 8e–h. Here, the LI method reduces the dark metal artifacts obviously, but it brings new streak-like dark artifacts and blurs the bone structure around the metals seriously. The NMAR and proposed methods protect the bone structures around the metals effectively and do not introduce new artifacts, but there are still some dark artifacts left. When compared with the NMAR algorithm, the proposed method has some improvements, there are less residual dark artifacts in the position as indicated by the arrow in Fig. 8h. Globally, in this dataset, the proposed algorithm performs better than LI and NMAR in suppressing metal artifacts. As mentioned above, this dataset has complicated structures, indicating that the uncorrected image harbors extremely severe metal artifacts, and the prior image quality will be poor. However, the best performance in this study proves the advantage of the proposed method for complicated bone and tissue structure images.

## Discussions

Metallic implants in the bodies of patients can lead to severe streaking artifacts, therefore degrading the quality of CT images and impacting the diagnostic procedure by obscuring image details. In this study, we propose a Gaussian diffusion sinogram inpainting algorithm to reduce the metal artifacts in a fan-beam scanner. In our work, the optimization objective is to minimize the prior energy. The Gaussian function is applied to diffuse prior energy, and the prior image is used to define the diffusion position and promote the sparsity of the original image. The gradient descent approach is applied to solve this problem. The performance of the proposed method is compared with two conventional MAR algorithms (LI, NMAR) using both simulation and clinical datasets. In the implementation of the proposed method and NMAR, we use the same prior image produced by the tissue-classified technique. From the results described in the last section, the proposed algorithm has a great advantage overall. However, there are still some factors impacting the effect of the proposed method.

First, the prior image quality is an important influential factor. In this study, the prior image is obtained by the tissue-classified method and often cannot introduce high-quality prior images because of inaccurate classification. Under this background, the proposed algorithm cannot produce an ideal corrected image at all times. This phenomenon reveals that the performance of the proposed method is highly dependent on the prior image. Thus, we may focus our next work on computing a high-quality prior image.

The second factor is the iteration numbers. The number of iterations determines the diffusion degree of the corrupted projections energy. Obviously, under the constraint of the prior image, when there is more energy diffused a better image quality will be obtained. The influence of the iteration times on the image quality is illustrated in Fig. 8.

**Fig. 9:**
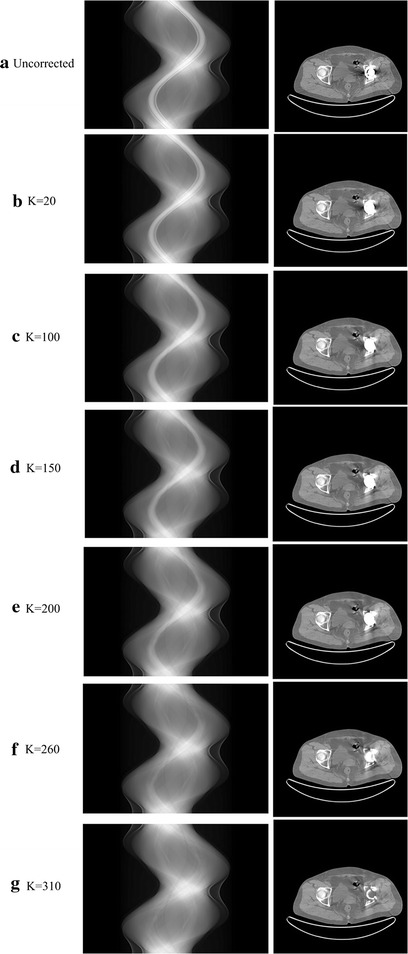
Comparison of sinograms and corrected images for different iteration times. The *left columns* are the sinograms, while the *right columns* are the corresponding images. **a** Original sinogram and uncorrected image. **b**–**g** Sinograms and corresponding corrected images of the iteration times 20, 50, 100, 150, 200, 260 and 310 (final result)

In Fig. 9, we chose the clinical hip dataset to explain the impact of the iteration numbers because this dataset has an obvious metal region in the projection domain, which is convenient to observe the change in the sinogram. From Fig. 9a–g, with an increase in iteration number we can easily find that the metal projection energy is gradually decreased, the metal artifacts existing in the corresponding images are decreased, and the image quality is improved. However, too many iterations will cause an increase in the correction time. Thus, in this work, to balance the image quality and corrected time, we set the stop criterion as ||*x* ^*k*+1^ − *x* ^*k*^||/||*x* ^*k*^|| < *η*, *η* = 1 × 10^−4^.

**Fig. 10:**
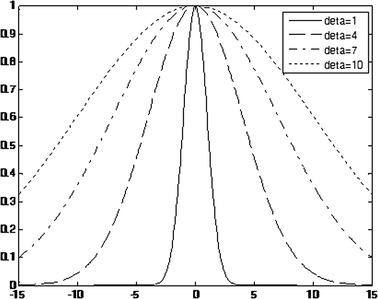
The Gaussian function curves for different *δ* values (deta in the picture)

**Fig. 11:**
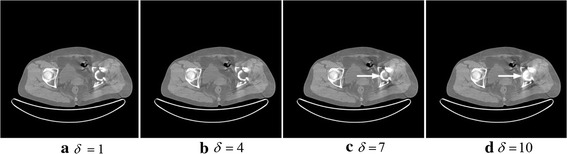
The results for different *δ* values. In **a**–**d**, the value of *δ* is set to 1, 4, 7, 10

The last key factor is the parameter *δ* in the Gaussian diffusion function, which determines the final diffusion extent and impacts the corrected image further. Figure 10 shows the Gaussian function with different *δ*. We can find that, as the value of *δ* becomes larger, the diffusion effect of the Gaussian function is better. Figure 11 shows the final corrected images for different *δ* values.

Figure 11 shows the corrected results for different *δ* values (1, 4, 7, 10). From the picture, Fig. 11a, b almost show the same results because the small *δ* values diffuse the energy slowly, and the optimal solution is easy to obtain. However, if the value of *δ* is too small, the iteration times would markedly increase. For example, when *δ* = 1, the iterative numbers would be *k* = 2000, which is a great waste of time. Conversely, as indicated by the arrows in Fig. 11c, d, the bone structure is not clear compared with (b). As mentioned before, the diffusion process can be observed as a gradient descent, so a large *δ* usually cannot diffuse energy to the minimum. Through numerous experiments such as this, we choose *δ* = 4.

## Conclusions

In this study, a Gaussian diffusion sinogram inpainting MAR algorithm is proposed, and the algorithm is realized via gradient descent. In this work, the prior image is obtained by a tissue-classified technique to improve the sparsity of the subtraction sinogram and to locate the diffusion position. The metal-only image is obtained by the simple thresholding method. The final corrected image is reconstructed by FBP after sinogram inpainting. The performance of the proposed algorithm is compared with that of the LI and NMAR algorithms using both simulation and clinical studies. Among the simulation and clinical studies, the proposed algorithm reduces the metal artifacts more effectively than the other two representative algorithms. The good performance of the proposed algorithm in several experiments proves that the method can be used in all types of clinical situations stably. However, the proposed algorithm relies on a prior image to some extent; thus, in our next work, we may use deep learning to compute high-quality prior images.

## Abbreviations

CT: computed tomography
FBP: filtered back-projection
MAR: metal artifact reduction
LI: linear interpolation
NMAR: normalized metal artifact reduction
LS: least squares
ART: algebraic reconstruction technique
POCS: projection onto the convex set
SNR: signal-to-noise ratio
NMAD: normalized the mean absolute deviation
ROI: region of interest
